# Mixtures of monodentate P-ligands as a means to control the diastereoselectivity in Rh-catalyzed hydrogenation of chiral alkenes

**DOI:** 10.1186/1860-5397-1-3

**Published:** 2005-08-26

**Authors:** Manfred T Reetz, Hongchao Guo

**Affiliations:** 1 Max-Planck-Institut für Kohlenforschung, Kaiser-Wilhelm-Platz 1, D-45470 Mülheim/Ruhr, Germany

## Abstract

The previously reported concept of using mixtures of monodentate ligands in a combinatorial manner in order to influence enantio- or regioselectivity of transition metal catalyzed processes has been extended to include diastereoselectivity. Accordingly, 1,2- and 1,3-asymmetric induction in the Rh-catalyzed hydrogenation of a chiral allylic alcohol and a chiral homo-allylic alcohol has been studied by using mixtures of monodentate P-ligands. It was found that appropriate 1:1 mixtures of two different P-ligands enhance the degree of diastereoselectivity relative to the use of the respective pure ligands themselves. Here, as in the previous cases regarding enantio- or regioselectivity, this type of combinatorial catalysis leads to improved catalytic profiles without the need to prepare new ligands.

The application of combinatorial chemical methods in asymmetric catalysis has emerged as a promising area of research, and indeed several reviews covering the subject have appeared.[1–8] It is based on the preparation of libraries of chiral metal complexes or metal-free catalysts[9] followed by medium- or high-throughput screening.[5] Catalyst diversity is achieved by the design and synthesis of modular ligands comprised of several building blocks which can be varied at will and easily assembled covalently. Another strategy involves the use of additives, catalyst poisons or catalyst activators.[10–11]

Based on the discovery that monodentate BINOL-derived phosphites,[12–13] phosphonites[14–15] and phosphoramidites [16–18] are excellent ligands in enantioselective Rh-catalyzed olefin-hydrogenation, we proposed in 2003 a new approach in the area of combinatorial asymmetric transition metal catalysis:[19–20] The use of mixtures of two chiral monodentate ligands L^a^ and L^b^ in metal catalysts of the type ML_2_, leading to two so-called homo-combinations and one hetero-combination which are in equilibrium with one another (Scheme 1). If the hetero-combination dominates due to enhanced activity and enantioselectivity, a superior catalytic profile can be expected. A mechanistic study of Rh-catalyzed olefin-hydrogenation using BINOL-derived monodentate phosphites (homo-combination) has shown that two such ligands are bound to the metal in the transition state of the reaction,[13] and it is certain that in the case of mixtures analogous species are involved.[19–20]

**Scheme 1:**
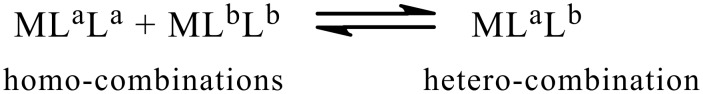


This new strategy was first employed in Rh-catalyzed olefin-hydrogenation using mixtures of BINOL-derived phosphites and phosphonites[19–20] and was then generalized.[21–23] Following our initial discovery,[19] Feringa and deVries reported related results using BINOL-derived phosphoramidites.[24] In all of these studies it was found that the mixture, and consequently the hetero-combination ML^a^L^b^, is more active and more enantioselective than either of the homo-combinations ML^a^L^a^ or ML^b^L^b^ which are also present in the reaction vessel.

In further research we discovered that it is also possible to employ a mixture composed of a chiral and an *achiral* monodentate P-ligand in asymmetric Rh-catalyzed olefin-hydrogenation, enhanced activity and reversal of enantioselectivity being observed in some cases.[25] Moreover, appropriate pairs of chiral and achiral monodentate P-ligands in other systems result in enhanced activity and nearly complete enantioselectivity.[26] Since it is currently not straightforward to predict which particular pair of chiral/chiral or chiral/achiral ligands is optimal for a given asymmetric transformation, a trial and error strategy is necessary.[27] Although this may not appear intellectually appealing, such an empirical process has an important advantage: *Once a library of monodenate ligands has put on the shelf by synthesis or commercial acquisition, simply mixing them pairwise in all possible permutational modes generates high catalyst diversity without the need to synthesize new ligands*. Of course, once a hit has been identified in the form of ML^a^L^b^, structural and mechanistic considerations may help in designing the optimal *bidentate* ligand composed of building blocks derived from the particular ligands L^a^ and L^b^.

We have recently extended this combinatorial principle beyond *enantioselectivity*[19–26] to include *regioselectivity*,[28] specifically in the hydroformylation of methacrylic acid *tert*-butyl ester. By using a 1:1 mixture of triphenylphosphine and a certain phosphinine (substituted phospha-benzene), 95% regioselectivity in favor of C-C bond formation at the higher substituted C-atom was achieved, whereas the use of the pure ligands as traditional homo-combinations led to low degrees of regioselectivity, but in the opposite sense.[28]

We now show for the first time that mixtures of monodentate P-ligands can also be used to influence *diastereoselectivity*, specifically in the Rh-catalyzed hydrogenation of chiral olefins. The literature contains several reports of 1,2- and 1,3-asymmetric induction in the hydrogenation of chiral olefins,[29–30] the underlying principle being Rh-complexation of functional groups present in the olefin (e.g., hydroxyl moieties) and/or 1,3-allylic strain[31] within the framework of substrate-directed stereoselectivity. Generally, a racemate was used to study diastereoselectivity. Catalyst ("reagent") control based on optically active ligands in the hydrogenation of chiral olefins has also been studied, in which case the substrates need to be used in enantiomerically pure form.[30] In some cases the role of the achiral ligands at the metal was studied, although not in an extensive manner. For example, Brown reported the Rh-catalyzed hydrogenation of the racemic allylic alcohol **1** with formation of diastereomers **2** and **3**, ketone **4** forming as a side product due to undesired isomerization.[29] From a small collection of mono-phosphines and bidentate diphosphines, diphos-4 led to the highest degree of diastereoselectivity in favor of the *anti*-product **2** (32:1). In our study we also chose this transformation as the model reaction. All reactions were performed with the racemate of **1**, although only one enantiomeric form is shown here for simplicity.

**Scheme 2:**
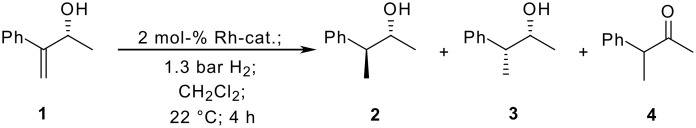


As a second model reaction the analogous Rh-catalyzed hydrogenation of the homo-allylic alcohol **5** with formation of diastereomers **6** and **7** was considered. Here again one enantiomeric form is shown arbitrarily, although a racemate was used.

**Scheme 3:**
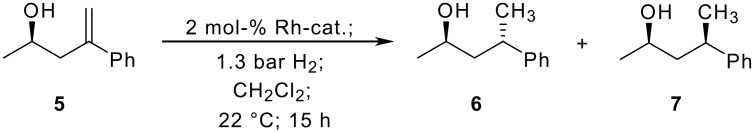


Monodentate P-ligands P1 – P23 were employed in the hydrogenation reactions, the Rh:ligand ratio being 1:2 in all cases. These ligands were not chosen on the basis of structural or mechanistic considerations; rather, they happened to be available in our laboratory, and indeed many of them are commercially available (P1–P6, P8, and P11–P14). Most of the ligands are achiral, but some contain one or even two stereogenic centers. Ligands P18, P20 and P21[32] were used in the (*S*)-form. In the case of the menthyl-derivative P15, a single stereoisomer was employed, although the configuration at phosphorus is unknown.[33] The menthol-derived ligand P23 having the (*S*)-configuration at phosphorus was described earlier.[34] Since the substrates **1** and **5** were used as racemates, kinetic resolution may be involved when the chiral ligands are employed, i.e., diastereoselectivity may change with conversion. This aspect was not a subject of the present study. In all hydrogenation reactions the standard precursor Rh(nbd)_2_BF_4_ (nbd = norbornadiene) was treated with the ligands to form pre-catalysts of the type RhL_2_(nbd)BF_4_ and free nbd. Dichloromethane served as the solvent.

**Scheme 4:**
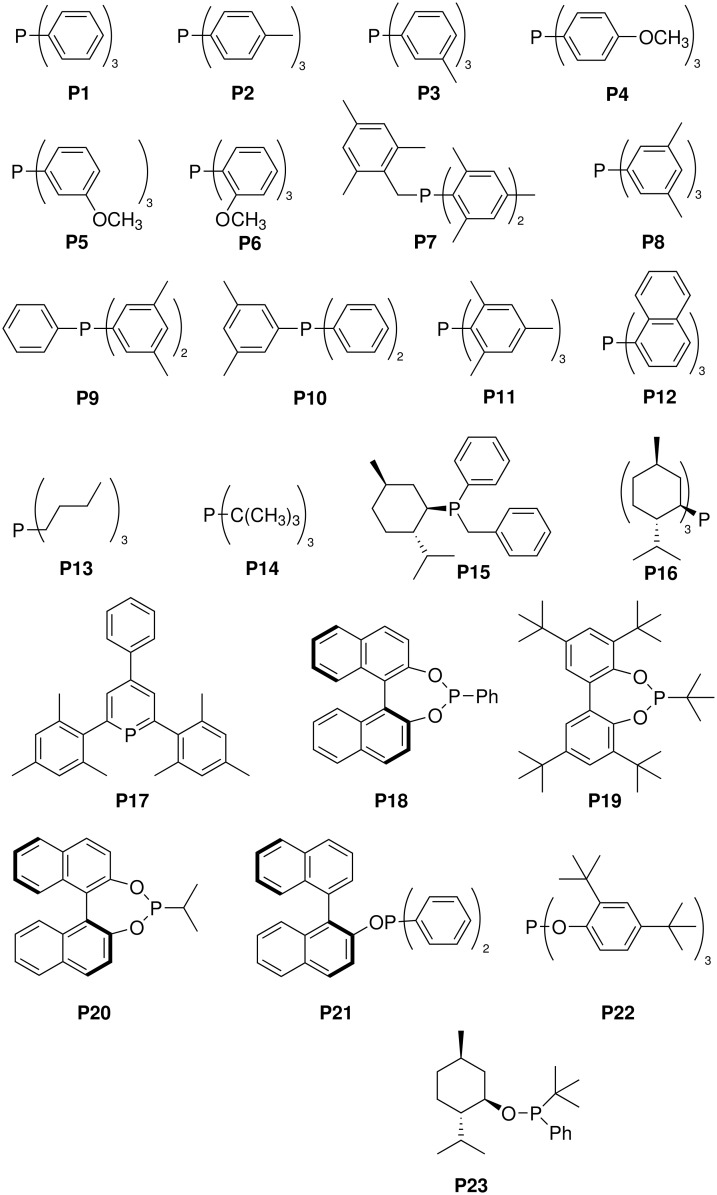


In the case of the hydrogenation of the allylic alcohol **1,** 21 of the 23 ligands were employed, which means a total of 210 different hetero-combinations in addition to the 21 conventional reactions (homo-combinations). Not all of them were actually tested. Rather, the combinatorial search was terminated after approximately 150 reactions had been performed in an arbitrary order. This random walk through a two-fold field of combinations led to the identification of 15–20 hits, i.e., those in which the hetero-combination results in a higher diastereoselectivity than either of the two homo-combinations themselves alone. Table 1 summarizes the essential part of the data. The hetero-combination P11/P17 results in the highest diastereoselectivity found in the total search, namely 27:1 in favor of the *anti*-product **2** (entry 33). This contrasts with the results obtained using the homo-combination P11/P11 which shows totally sterorandom behavior (entry 11), and the other homo-combination comprising the phosphinine P17/P17[35] which results in a diastereoselectivity of only 5:1 (entry 17). Another noteworthy catalyst system is composed of the bulky phosphite P22. This ligand alone delivers a moderate diasteroselectivity of 4:1 (entry 20), whereas various hetero-combinations based on P22 and other P-ligands induce markedly enhanced selectivities (entries 23, 25, 26, 27, 28, 29, 30, 32, 34, 36). It is of no surprise that many hetero-combinations fail to induce significant improvements (data not shown), and that some result in diastereoselectivities which are actually lower than the respective homo-combinations. An example of the latter is hetero-combination P18/P22 leading to a **2**:**3** ratio of only 2:1, while P18/P18 and P22/P22 are both more selective (each 4:1; entries 18 and 20, respectively).

**Table 1:** Diastereoselective hydrogenation of the allylic alcohol **1**.

Entry	Ligand	Conversion (%)	Diastereoselectivity **2**:**3**	Side product **4** (%)

*Homo-combinations*				

1	P1/P1	100	10:1	47
2	P2/P2	100	17:1	33
3	P3/P3	100	14:1	51
4	P4/P4	100	16:1	30
5	P5/P5	100	10:1	49
6	P6/P6	7	2:1	4
7	P7/P7	78	1:1	8
8	P8/P8	100	13:1	64
9	P9/P9	100	16:1	58
10	P10/P10	100	14:1	47
11	P11/P11	93	1:1	7
12	P12/P12	44	3:1	27
13	P13/P13	100	17:1	15
14	P14/P14	74	2:1	8
15	P15/P15	100	12:1	1
16	P16/P16	79	1.5:1	18
17	P17/P17	100	5:1	98
18	P18/P18	51	4:1	15
19	P20/P20	64	4:1	24
20	P22/P22	45	4:1	7

*Hetero-combinations* (hits)				

21	P1/P12	100	19:1	45
22	P1/P15	100	18:1	24
23	P1/P22	100	17:1	45
24	P2/P12	100	21:1	40
25	P2/P22	100	19:1	33
26	P4/P22	100	18:1	32
27	P5/P22	100	18:1	47
28	P6/P22	25	8:1	6
29	P8/P22	100	20:1	65
30	P9/P22	100	20:1	59
31	P10/P15	100	20:1	30
32	P10/P22	100	19:1	50
33	P11/P17	56	27:1	48
34	P12/P22	74	9:1	4
35	P17/P20	100	7:1	70
36	P17/P22	100	8:1	56

Diastereoselectivity in the hydrogenation of the homo-allylic alcohol **5** involves 1,3-asymmetric induction, which is usually considered to be more difficult.[30–31] In this case the combinatorial search was restricted to ligands P1-P17, P19 and P21–P23, although again not all permutations were actually tested. Table 2 reveals the diastereoselectivities resulting from the use of the homo-combinations and of those hetero-combinations which exert a substantial stereochemical influence (hits). Inspection of the best results arising from the homo-combinations shows that diastereoselectivity in favor of the *anti*-product **6** is moderate (e.g., entries 6, 12, 16, 19, 20). Since in the present study the racemic form of **5** was employed, the degree of "reagent control" in the case of the chiral ligands P16, P18 and P20 was not ascertained. The highest *syn*-selectivity favoring **7** amounts to only 1:1.8 when using ligand P4 (entry 4). From the list of hetero-combinations showing a stereochemical influence relative to the use of the respective pure ligands, three lead to substantial *syn*-selectivity (**6**:**7** = 1:5), namely P4/P23, P9/P23 and P10/P23 (entries 24, 29 and 31, respectively). In all three cases the chiral phosphinite **23** is involved, which as a homo-combination actually favors the opposite diastereomer (**6**:**7** = 3:1; entry 21). With regard to maximizing *anti*-selectivity, the hetero-combination P7/P21 results in a diastereoselectivity of 18:1 in favor of **6** (entry 26) which is clearly better than P21 alone or any other homo-combination listed in Table 2.

**Table 2:** Diastereoselective hydrogenation of the homo-allylic alcohol **5**.

Entry	Ligand	Conversion (%)	Diastereoselectivity **6**:**7**

*Homo-combinations*			

1	P1	100	1:1.4
2	P2	100	1:1.5
3	P3	100	1:1
4	P4	100	1:1.8
5	P5	100	1.2:1
6	P6	100	7:1
7	P7	6	3:1
8	P8	100	2:1
9	P9	100	1:1
10	P10	100	1:1
11	P11	2	2:1
12	P12	100	7:1
13	P13	100	1.2:1
14	P14	1	1.6:1
15	P15	100	2.6:1
16	P16	100	9:1
17	P17	32	3:1
18	P19	89	3:1
19	P21	2	6:1
20	P22	100	10:1
21	P23	100	3:1

*Hetero-combinations* (hits)			

22	P3/P19	100	1:2
23	P4/P19	100	1:2.6
24	P4/P23	100	1:5
25	P5/P19	100	1:1.8
26	P7/P21	26	18:1
27	P8/P19	100	1:1.8
28	P9/P19	100	1:2
29	P9/P23	100	1:5
30	P10/P19	100	1:2
31	P10/P23	100	1:5
32	P11/P14	14	6:1
33	P11/P15	100	7:1
34	P14/P15	86	9:1
35	P14/P19	77	5:1
36	P17/P19	100	1:3

The purpose of the present study was to demonstrate that mixtures of two monodentate P-ligands can influence the diastereoselectivity of transition metal catalyzed reactions of chiral substrates. This was accomplished by studying the diastereoselective Rh-catalyzed hydrogenation of the chiral allylic alcohol **1** and the homo-allylic alcohol **5**. Since the goal was proof-of-principle, only 23 randomly chosen monodentate P-ligands that happened to be available in our laboratory were employed, and not even all combinations were actually tested. We do not claim that the hits reported here constitute the optimal catalyst systems. If in the present reactions (or in other processes) higher diastereoselectivities are strived for, the combinatorial search needs to be systematized and the library of ligands extended. Indeed, numerous analogs of the present ligands are known, many of which are commercially available. Moreover, important classes of P-ligands not considered here can also be included in future studies of this kind.

At this time we refrain from proposing any hypotheses regarding the structural and mechanistic reasons for enhanced diastereoselectivity when using certain combinations of monodentate P-ligands. The same applies to the observed differences in rate. The combinatorial search is purely empirical. However, in addition to the inherent different degrees of diastereoselectivity arising from the homo- and hetero-combinations, their relative amounts present in catalytically active form and the relative reaction rates dictate the stereochemical outcome. Since the relative amount of catalytically active forms is thermodynamically controlled, changing the L^a^ : L^b^ ratio may affect diastereoselectivity and can thus be used as a tool in future studies. It also remains to be seen if mixtures of chiral ligands affect diastereoselectivity in reactions of chiral substrates when they are used in enantiomerically pure form.

In conclusion, the previous concept of using mixtures of monodentate ligands in order to influence enantio-[19–26] and regioselectivity [28] of transition metal catalyzed reactions has been extended to include diastereoselectivity. The idea of using mixtures of monodentate ligands in a combinatorial manner is rapidly emerging as a powerful method to enhance activity and selectivity of transition metal-catalyzed reactions.
